# Spontaneous Rupture of the Omental Artery Treated by Transcatheter Arterial Embolization

**DOI:** 10.1155/2012/273027

**Published:** 2012-12-17

**Authors:** Masamichi Takahashi, Yujiro Matsuoka, Tsuyoshi Yasutake, Hiroyuki Abe, Kazuhiro Sugiyama, Kazuyuki Oyama

**Affiliations:** ^1^Department of Radiology, Tokyo Metropolitan Bokutoh Hospital, 4-23-15 Kotobashi, Sumida, Tokyo 130-8575, Japan; ^2^Department of Emergency and Critical Care Medicine, Tokyo Metropolitan Bokutoh Hospital, 4-23-15 Kotobashi, Sumida, Tokyo 130-8575, Japan

## Abstract

Intra-abdominal hemorrhage caused by omental artery rupture is a rare condition. There are few reports on the treatment of omental artery rupture with only transcatheter arterial embolization (TAE). A 27-year-old man presented to our emergency room with upper abdominal pain that suddenly occurred during sleep. Abdominal computed tomography (CT) revealed fluid collection in the peritoneal cavity and a left subphrenic hematoma with extravasation. Celiac angiography revealed extravasation from the omental artery, which arose from the proximal left gastroepiploic artery. A microcatheter was advanced into the left gastroepiploic artery and around the culprit artery bifurcation, which was embolized by inserting coils. The postoperative course was uneventful without worsening of anemia or abdominal symptoms. The patient was discharged after the absence of extravasation was confirmed by contrast-enhanced CT. Although surgical therapy has often been performed for omental bleeding, TAE, which is less invasive and has the advantage of simultaneous diagnosis and treatment, should be attempted as the first-choice therapy.

## 1. Introduction

Rupture of the omental artery is a relatively rare condition that can lead to life-threatening intra-abdominal hemorrhage [1]. Causes of omental artery rupture include penetrating or blunt abdominal trauma, neoplasia [2], arterial aneurysm rupture [3], and complications of anticoagulant therapy [4]. However, it rarely develops without any specific triggers [5, 6]. We found only four reports on transcatheter arterial embolization (TAE) for rupture of the omental artery [7–10]. Along with a review of these reports, we herein present a case of omental artery rupture treated by TAE in a young patient who had no history of a related disease or trauma.

## 2. Case Presentation

A 27-year-old man presented to our emergency unit after having been woken from sleep at midnight because of severe abdominal pain. His medical and familial histories were unremarkable. His vital signs and laboratory evaluation results, including the hemoglobin level (15.2 g/dL), showed no abnormalities. He was suspected to have gastritis and was discharged with an H2-blocker. However, the abdominal pain did not improve. It worsened when stretching his back and breathing. He returned to our hospital because of temporary loss of consciousness when rising from his bed the next morning. 

**Figure 1:**
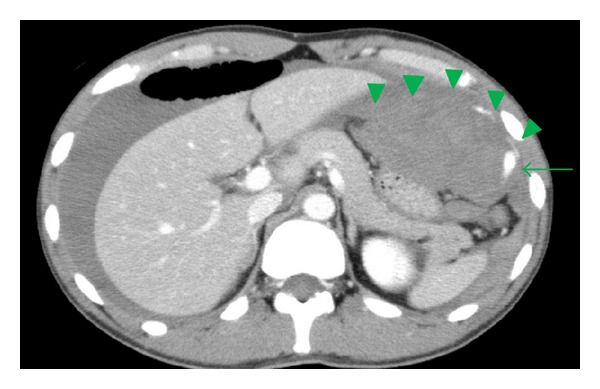
Abdominal enhanced CT revealed a hematoma (arrows) with extravasation (arrowheads) located in the left part of the omentum and relatively high-attenuation fluid in the abdominal cavity.

Contrast-enhanced CT revealed high-density ascites retention that was thought to contain blood. There was a left subphrenic hematoma compressing the stomach and spleen with extravasation (Figure 1). Omental bleeding was suspected based on these findings. After the CT scan, a blood test showed worsening anemia (hemoglobin, 8.6 mg/dL). His blood pressure was 132/60 mmHg, and his heart rate was 112 beats/min. Consequently, a packed red blood cell transfusion was started, and urgent angiography was performed to obtain a diagnosis and begin appropriate treatment.

**Figure 2:**
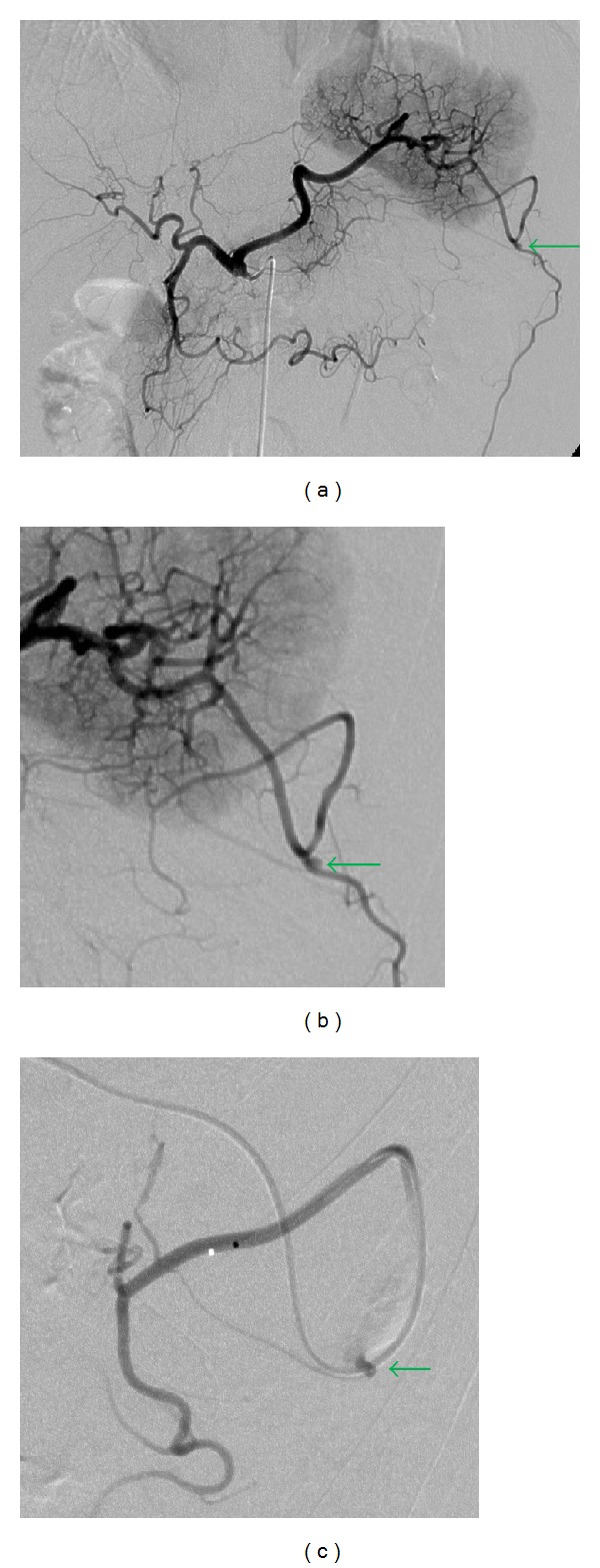
Celiac and left gastroepiploic arteriography revealed active extravasation (arrow) of the omental artery arising from the left gastroepiploic artery (a, b, c).

**Figure 3:**
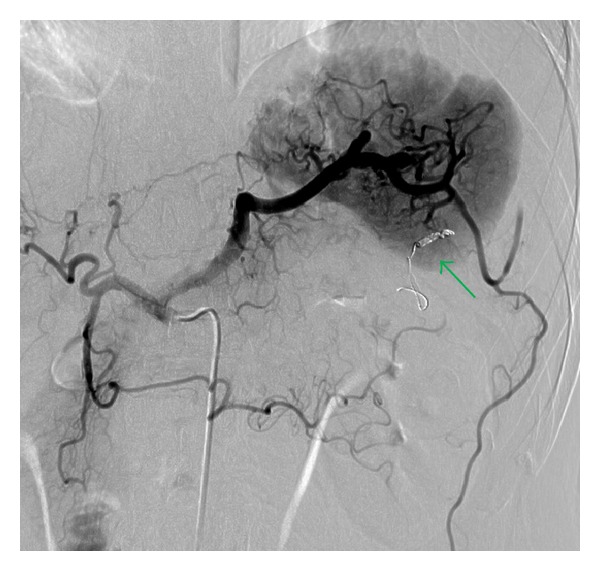
Celiac arteriography after embolization revealed arrested bleeding from the omental artery. Microlocoils are placed in a left gastroepiploic artery (arrow).

After the insertion of a 5-F sheath introducer from the right femoral artery under local anesthesia, a 5-F shepherd hook catheter (Terumo Clinical Supply, Tokyo, Japan) was advanced through the sheath introducer with its tip positioned in the celiac artery. Celiac arteriography revealed bleeding from the omental artery, which arose from the left gastroepiploic artery (Figure 2). The left gastroepiploic artery arose from the splenic artery. Superselective catheterization of the left gastroepiploic artery through the splenic artery was then performed with a 2.0-F microcatheter (Sniper 2-*μ*7; Terumo Clinical Supply, Tokyo, Japan), which was coaxially advanced through the 5-F catheter as distally as possible into the left gastroepiploic artery. Embolization with microcoils was performed. A 4 mm diameter, 6 cm long Interlock Fibered IDC Occlusion System (Boston Scientific, Natick, MA, USA) and a 2 × 4 mm Tornado Embolization Coil (Cook, Bloomington, IN, USA) were used as microcoils. After the embolization, the procedure was completed after confirming the disappearance of extravasation by angiography performed from the left gastroepiploic and celiac arteries (Figure 3).

The patient's abdominal pain was relieved, and progression of anemia was not observed after the procedure. Two days after the procedure, contrast-enhanced CT revealed no extravasation. No complications or rebleeding occurred, and he was discharged on the 10th hospital day.

## 3. Discussion

Rupture of the omental artery is a rare condition that can cause intra-abdominal hemorrhage [1]. Several causes of omental bleeding are known, including injury and irritation from trauma, neoplasia [2], arterial aneurysm rupture [3], and anticoagulant therapy [4]. However, it rarely develops spontaneously [5–7]. In our case, there was no episode of abdominal trauma or bleeding disorders and no appreciable familial or medical history.

The age of onset of spontaneous rupture of the omental artery ranges widely from the 20 s to 80 s [5–11]. It occurs more frequently in men than in women, with a male-to-female ratio of 6 : 1. It frequently begins with epigastric pain and occasionally involves digestive symptoms including nausea, vomiting, and diarrhea. Treatment results in a good prognosis. However, because of two reported cases of rebleeding during the follow-up period after conservative management, aggressive treatment is preferable [11]. 

Matsuda reviewed 37 cases of spontaneous rupture of the omental artery reported from 1986 to 2010 in Japan [11]. According to the report, 33 of 37 cases underwent surgery, including 29 cases of omentectomy, 2 of hematoma removal, and 2 of ligation, all of which achieved hemostasis. 

However, there have been few reports on TAE for spontaneous rupture of the omental artery, including only one case reported in English and three cases in Japanese [7–10]. No cases were associated with triggers such as bleeding factors or injury. Omental artery bleeding was suspected based on CT findings, and the bleeding site was confirmed by angiography. Microcoils or a combination of microcoils and a gelatin sponge were used for embolization. Although three cases were successfully managed by TAE, the remaining case developed rebleeding 2 days later and underwent omentectomy [10]. No serious treatment-related complications were reported.

One of the reasons that surgical treatment has been frequently performed for omental bleeding is that very few cases have been correctly diagnosed before treatment. According to the report by Matsuda [11], as many as 17 cases (46%) were surgically treated because of preoperative misdiagnosis as acute abdomen attributed to acute appendicitis, perforation of the digestive tract, or intra-abdominal abscess, while only 7 cases (17%) were correctly diagnosed as omental bleeding before treatment. This result suggests that TAE might not be considered as a treatment option in many other cases. Another reason is that although reports published before interventional radiology became widely used comprise a high proportion of the reviewed reports, all four of the above-mentioned cases that underwent TAE were reported after 2008 (two cases reported in 2008, one in 2009, and one in 2011).

TEA is less invasive than surgical treatment and has the advantage of simultaneous diagnosis and treatment. The omental arteries arising from the left and right gastroepiploic arteries are anastomosed to each other in the periphery. The omental artery does not provide the main blood supply to organs other than the omentum. Therefore, TAE for the omental artery is considered to be a procedure with a low risk of major emboli in other organs. Serious complications were not observed in the four cases treated with TAE. 

However, TAE should be carefully performed in the case of proximal embolization because of the possibility of rebleeding through the collateral circulation [10]. When a microcoil is used as embolic material, it is ideal to perform isolation by inserting a microcatheter to the distal site of bleeding. However, it may be difficult to bring it to the bleeding point in the case of bleeding from a tiny vessel. In our case, the absence of a collateral route that anastomosed with the culprit vessel was confirmed before the embolization. Coil embolization was then performed from proximal to distal by getting the coil to straddle the bifurcation of the culprit vessel because the culprit vessel was too thin to be selected. N-butyl 2-cyanoacrylate (NBCA) (Histoacryl Blue; Braun, Melsungen, Germany), which was not used in our case, should be considered as embolic material in the case of bleeding from a tiny branch vessel when a microcatheter is unavailable. When mixed with iodized oil (Lipiodol; Laboratoire Guerbet, Roissy, France) in the appropriate ratio, NBCA, which is a liquid and permanent embolic agent, can reach sites of arterial bleeding that a microcatheter cannot [12].

Absence of bleeding from a collateral route should be confirmed by angiography not only from one side of the gastroepiploic artery, but also from the root of the celiac artery. If TAE does not result in complete embolization, placing a microcoil in the artery adjacent to a bleeding site may provide an indication of the bleeding site when surgical treatment is subsequently performed. 

## 4. Conclusion

TAE successfully stopped the bleeding of the omental artery that developed in a young patient with no specific trigger. Because TAE for the omental artery is an easy technique with a low risk of complications, it should be attempted as the first-choice therapy.
